# Feeding Practices of Infants and Toddlers by Their Mothers in Selected Northern Emirates of the United Arab Emirates

**DOI:** 10.3390/nu14183719

**Published:** 2022-09-09

**Authors:** Habiba I. Ali, Emmanuella Magriplis, Amita Attlee, Ayesha S. Al Dhaheri, Leila Cheikh Ismail, Lily Stojanovska

**Affiliations:** 1 Department of Nutrition and Health, College of Medicine and Health Sciences, United Arab Emirates University, Al Ain 15551, United Arab Emirates; 2 Department of Food Science & Human Nutrition Laboratory of Dietetics and Quality of Life, Agricultural University of Athens, 11855 Athens, Greece; 3 Department of Clinical Nutrition and Dietetics, College of Health Sciences, University of Sharjah, Sharjah 27272, United Arab Emirates; 4 Nuffield Department of Women’s & Reproductive Health, University of Oxford, Oxford OX1 2JD, UK; 5 Institute of Health and Sport, Victoria University, P.O. Box 14428, Melbourne, VC 8001, Australia

**Keywords:** feeding practice, infants, toddlers, breastfeeding, complementary feeding, United Arab Emirates

## Abstract

Research on the feeding practices of infants and young children in the United Arab Emirates (UAE) is limited, especially in the northern regions of the country. A retrospective web-based survey was conducted to assess child feeding practices among the mothers of young children aged 6 months to 2.5 years in the northern emirates of the UAE. Information from a total of 475 mothers was collected on maternal socio-demographic factors, child feeding practices, and the use of vitamin and mineral supplements. For the first 6 months, 46.7% of the infants were exclusively breastfed, 43.8% were fed on both breastmilk and formula, and 9.5% were given formula only. Significant differences in the types of feeding were found correlating with maternal age (*p* = 0.02) and employment status (*p* < 0.001) but not with educational level, with a higher proportion of younger and unemployed women exclusively breastfeeding. However, although a significantly higher proportion of mothers with lower educational levels breastfed their children for ≥6 months (*p* = 0.026), they introduced “other milk” (non-breastmilk or formula) before the child reached the age of 12 months (*p* = 0.022). In this study, 22.1% of the infants and 8.1% of the toddlers did not receive an animal source of iron, while 52.6% of the children received vitamin/mineral supplements. The median daily frequency intake of sweets and savory snacks was substantially higher than the respective intake of fruits and vegetables. Intervention programs that focus on healthy infant and toddler feeding, including food sources of iron and nutrient-dense food groups, are needed in the UAE.

## 1. Introduction

Optimal nutrition is essential for the growth, wellbeing, and development of children, especially in the critical period from birth to two years of age [1]. Emphasis is placed on the first 1000 days of life due to the infant’s rapid growth and neurodevelopmental changes [2]. Moreover, the optimal feeding of infants and young children during the first two years of life decreases morbidity and mortality and lowers the risk of chronic diseases in adulthood [3,4]. The World Health Organization (WHO) and the United Nations International Children’s Fund (UNICEF) recommend exclusive breastfeeding (EBF) for the first 6 months of the child’s life and continued breastfeeding up to two years of age [1]. Healthy weaning with nutritionally sufficient and age-appropriate complementary foods should ideally be offered after 6 months of age [1,5]. However, according to WHO, only 44% of infants aged 0–6 months are exclusively breastfed [1].

The United Arab Emirates (UAE) is an economically advanced country with developments in many fields, including infrastructure, trade and economy, and social and human development. The UAE health authorities have consistently made efforts to promote and support breastfeeding [6], and the country has adopted the WHO recommendation of EBF and the timely initiation of age-appropriate complementary feeding [1]. Despite these efforts, previous studies in the UAE reported lower rates of exclusive breastfeeding and adherence to the recommended introduction of complementary foods at 6 months [7,8,9]. Research conducted in the UAE also highlighted the need to understand the possible barriers related to maternal employment, age, and education, which seem to influence breastfeeding patterns [7,8,9,10,11], and develop applicable strategies to promote and support breastfeeding [8,9,10]. Studies in the Middle East [12,13,14,15] and globally [16,17,18,19,20,21] also identified maternal age, education, and employment status as factors that affect breastfeeding.

Adequate iron intake is important for the growth and development of young children [22,23] and the prevention of iron deficiency anemia [24], which is one of the most prevalent nutritional deficiencies globally. According to the WHO, approximately 42% of children who are less than 5 years of age globally have iron deficiency anemia [25]. Although the UAE is economically advanced, studies conducted in the UAE have reported a high prevalence of iron deficiency anemia [26,27]. The low intake of iron-containing foods is considered to be the leading cause of anemia in young children [24]. In the Eastern Mediterranean Region, inadequate micronutrient intakes, including iron, were found to be prevalent [28]. From the age of 6 months, all infants and toddlers should receive iron-rich complementary foods, including meat products and/or iron-fortified foods. However, studies in the UAE have reported the delayed introduction of iron-rich animal protein in infants [9] and an inadequate typical dietary intake of iron among infants and toddlers [29,30].

Infants and toddlers need to consume a variety of nutrient-dense foods to meet their energy and nutrient needs and support their rapid growth and development. A number of studies have evaluated the feeding practices of infants and toddlers in the UAE [8,9,29,30,31]. A recent study conducted in three emirates of the UAE found that a high proportion of toddlers aged 12–23.9 months did not consume the recommended amounts of vegetables, fruits, and lean meats/beans (93%, 87%, and 54%, respectively) [30]. Moreover, most of the previous research has been conducted in three of the seven emirates (Abu Dhabi, Dubai, and Sharjah) in the UAE. Therefore, this study focused on the other four emirates in the northern region (Ajman, Al Fujairah, Ras Al-Khaimah, and Umm Al Quwain), from which less information about child feeding practices has been reported. This study aimed to assess the feeding practices of infants and toddlers aged 0.5–2.5 years by their mothers, including breastfeeding, solid foods, and beverages, as well as the introduction of food sources of iron. In addition, maternal socio-demographic factors associated with child feeding practices, including age, educational level, and employment status, were considered. The study was conducted as part of a larger project examining the role of diets and food systems in the prevention of overweight, obesity, and non-communicable chronic diseases in the UAE.

## 2. Materials and Methods

### 2.1. Design and Participants

A retrospective survey was carried out between August 2020 and March 2021. The inclusion criteria were mothers with children aged 6 months to 2.5 years, UAE nationals, and non-nationals residing in any of the following emirates in the northern region: Ajman, Umm Al Quwain, Ras Al Khaimah, and Al Fujairah. These four emirates were chosen due to the limited research on child feeding practices and dietary intakes in these areas. We included mothers of infants aged from 6 months upwards in order to examine their adherence to the EBF recommendations. We included toddlers aged 1–2.5 years (instead of 1–3 years) since, after 2.5 years, toddlers can start preschool and, therefore, their dietary habits may be altered.

To complete the study questionnaire, participants were expected to be fluent in either English or Arabic. The study power (80%) was calculated based on the number of births in the four emirates during the year 2017, which was the latest birth data available on the website of the UAE’s Federal Competitiveness and Statistics Authority in 2020, when the study was conducted. In 2017, the total number of births in the four emirates was 16,679 [31]. The sample size calculation was performed using the formula:

$$n=Z^{2}\frac{P\left(1-P\right)}{e^{2}}$$

where:

*e* = 0.05, the desired level of precision (i.e., the margin of error).

*P* = 0.5, as guided by the STEPS process, when being conservative about estimating the proportion of the population that has the attribute in question. Hence, q is 1–*p* = 0.5.

The *Z* Value at the 5% level set for this study is equal to 1.96 (for a 95% confidence interval); n (sample size) = 385.

Therefore, a sample of 385 live births in our target population was deemed sufficient to provide the required confidence level. Including a 10% attrition rate, the total sample required for the 80% study power resulted in 423 individuals.

Emails with a link to the participant information sheet were sent to UAE university students, informing them of the research project and encouraging them to assist in recruiting family, friends, and community members that met the predetermined inclusion criteria (snowball design). To increase the randomization and minimize selection bias associated with snowball sampling, messages containing online promotion of the survey were sent through social media (including WhatsApp™, frequently used by this population) to undisclosed recipients lists to inform the targeted community and increase participation in the survey. Once participation was confirmed, an email containing a link to the consent-to-participate form, as well as the questionnaire, were shared. Contact details of the research assistants were provided to participants in case they required any guidance when completing the survey. All data were collected anonymously.

The study was approved by the United Arab Emirates University Social Sciences Research Ethics Committee, Al Ain, United Arab Emirates (Protocol #ERS_2020_6083). All procedures followed the principles of the Declaration of Helsinki.

### 2.2. Measures

The survey used two online questionnaires adapted from the Child Feeding Questionnaires of the population-based, multi-country INTERGROWTH-21st Project [32,33,34]. One questionnaire was formulated for mothers of 6–12-month-old infants and another for mothers of 1–2.5-year-old children (toddlers). Mothers with children in both age groups were instructed to complete the survey only for the infant. Each questionnaire consisted of the following main sections: (1) maternal socio-demographics (total of 6 questions); (2) child feeding practices (total of 24 questions); and (3) the use of vitamin and mineral supplements (1 question). Child feeding questions covered exclusive breastfeeding, complementary foods, the introduction of animal-based protein foods (other than milk), the introduction of “other milk” and honey, and who feeds the child. The “other milk” includes cow’s milk, soya milk, and camel milk. The mothers were asked if they knew about food sources of iron (with the possible answers of “Yes” and “No”). They were also asked if vitamin and mineral supplements were given to the child and about the types of the supplement given.

For the 6–12-month age group, the INTERGROWTH-21 Infant Feeding Questionnaire for infants during the first year of life and a 39-item qualitative food frequency questionnaire (FFQ) were used to assess the food intake patterns during the past 28 days [33,34]. For the 13–30-month age group, the INTERGROWTH-21 2-Year Visit Food Intake Questionnaire was used to assess general child feeding practices and feeding patterns during the past 28 days based on a 39-item qualitative FFQ [33,34]. A qualitative FFQ, otherwise called the Food Propensity Questionnaire, is an FFQ introduced in 2006, which is used in nutritional epidemiology to assess the frequency of food intake solely and provide valuable information about the long-term intake and variation in food group consumption, which this study aimed to examine [35]. Portion size was not included, since this questionnaire was not used to estimate energy and macronutrient intakes. The infant and toddler FFQ provided 6 frequency choices, without food portions (never, 1–3 times/month, 1–3 times/week, >3 times/week, 1–3 times/day, and >3 times/day). The FFQs were modified to be culturally adapted to the UAE population. For example, food items given to young children, such as honey and camel milk, which are traditionally consumed in the UAE, were added to the questionnaires [7]. The list of the foods included in the infant and 2-year FFQs are available in the Supplementary Materials (Table S1-Food Frequency Questionnaire). Furthermore, a study among toddlers in the UAE reported the low intake of food sources of iron, such as lean meats and beans [30]. Since animal proteins (excluding milk) are better sources of iron compared to foods of plant origin, mothers were asked about the types of food sources of iron, such as meats and chicken, that were introduced into their children’s diets and their timing. Questions related to animal of sources of iron were adapted from previous research [36]. The Arabic translation of the questionnaire was independently conducted by two members and reviewed by a third member of the research team. The questionnaires were back-translated into English by the latter member to assure that the original meaning of the items was retained. This was followed by pilot-testing on a sample of 15 mothers before applying it to the study so as to assure clarity and understanding.

The data collection period was from August 2020 to March 2021. Maternal age was categorized into 18–25 years, 26–33 years, and 34 years and above. The educational level of the mothers was categorized as high school or less and college/university. Participants were grouped as employed or unemployed.

In the qualitative FFQ, breastmilk, formula, other animal milk, and water were calculated according to daily intake while fruit juices and soft drinks were calculated according to weekly intake. Food groups were analyzed according to either daily or weekly intake. Grains and cereals, dairy, fruits, and vegetables are presented according to daily intake. Foods such as eggs, legumes, meat, sweets, and snacks were calculated based on weekly consumption.

### 2.3. Statistical Analysis

Categorical data were presented as frequencies and percentages (n, %). Continuous data were screened for extreme outliers and potential misclassifications by tabulation techniques and the use of scatter plots. Kernel density and P-P plots were used to examine the distribution. Generally, distributed variables were summarized using means and standard deviations (SD), whereas medians and interquartile ranges (IQR: 25–75th % -ile, to avoid extreme variables) were used to describe the distribution of skewed data and were presented as the total and by group (age group, maternal employment status, educational level, and knowledge of food sources of iron). Student’s *t*-test (for normally distributed data) or Kruskal–Wallis tests (for skewed data) were used to examine the group differences. When more than 2 group categories were examined, a one-way analysis of variance (ANOVA) was performed. Relative frequencies were calculated for the categorical data, and the chi-squared test or Fisher’s exact test (for small sub-samples; n < 5) were used to examine the between-group differences detected. Multinomial logistic regression was used to assess the effects of age, educational level, and employment status according to the type of food given to the infant prior to 6 months (breastmilk and formula compared to EBF, and formula only compared to EBF). The model was further adjusted for known a priori confounding variables (nationality, age of gestation, type of delivery method). The statistical analysis was performed using Stata 14.0 [37]. A *p*-value of < 0.05 was considered significant.

## 3. Results

### 3.1. Maternal Socio-Demographic Characteristics

The socio-demographic maternal characteristics of the study participants are presented in Table 1. A total of 475 mothers completed the survey. The participants were 253 mothers of children aged 6 months to 12 months (infants) and 222 mothers of children aged 1–2.5 years (toddlers) living in the four emirates in the northern region of the UAE. Most of the participants (47.8%) were in the age group of 26 to 33 years, and the majority (70%) had completed university education. Similarly, most (72%) of the women were employed, either part-time or full-time. Fifty-five percent of the participants were UAE nationals and the remaining were of different nationalities.

### 3.2. Types of Milk Given to Infants and Toddlers

**Figure 1:**
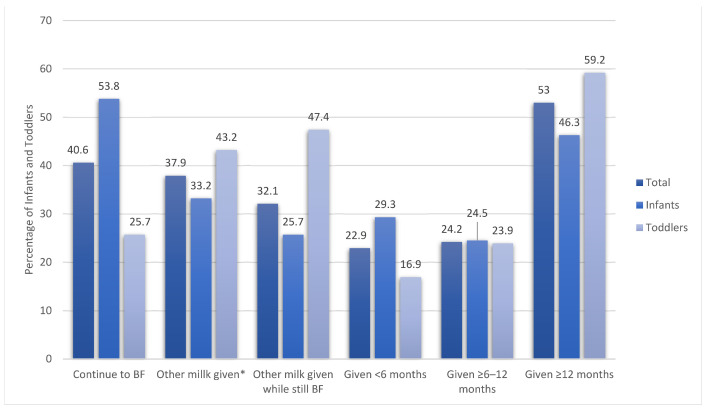
Feeding practices according to milk type among infants and toddlers. * Non-breastmilk or formula milk; BF = breastfed.

Most of the mothers (31.2%) relied upon information from health professionals for feeding their children, followed by family and friends and social media (28.8 and 27.6%, respectively) (Table 1). Figure 1 shows the types of milk (breastmilk, non-breastmilk, or formula) feeding practices of infants and toddlers in the study. Almost 41% of the total infants and toddlers enrolled in this study were being breastfed (53.8% of infants and 25.7% of toddlers). Most of the infants and toddlers received “other milk” at or after 1 year of age (53%), and almost one-third (33.2%) of the infants were given “other milk”, such as non-breastmilk or formula. An analysis of the introduction of other milk before 6 months of age showed that a higher proportion of infants than toddlers were given other milk (29.3% of toddlers vs. 16.9% of infants, *p* = 0.032) (Figure 1).

### 3.3. Maternal Socio-Demographic Characteristics and Feeding Practices of Infants and Toddlers

The child feeding practices according to the maternal age category are shown in Table 2. Significant differences were found in the types of feeding for the first 6 months according to the mother’s age (*p* = 0.02). The majority, or 58.5%, of the mothers aged between 18 and 25 years fed their infants only breastmilk compared to 40.9% of the mothers who were older than 33 years, while mothers older than 33 years used both breastmilk and formula (51.4% vs. 30.2%), respectively. Overall, 46.7% of mothers exclusively breastfed their infants for the first 6 months, while 43.8% used both breastmilk and formula, and 9.5% of mothers used formula. Of those who breastfed, 46.7% continued to BF for ≥6 months. Overall, 41.6% of the mothers reported the introduction of solid food before the child was 6 months old.

There was no significant difference in terms of the age category in which mothers introduced food sources of animal protein, while significant differences were found in the types of first animal protein that the children were fed with (*p* = 0.017). A total of 17% of mothers reported that they had not introduced any animal protein (23.5% of the infants and 5.4% of the toddlers).

The results showed a significant difference in the provision of vitamin and mineral supplements to children according to the mother’s age (*p* = 0.01). A lower proportion (44.3%) of mothers in the age group of 18–25 years gave supplements to their children compared to those aged 26 years and older. Specifically, 59% of those aged between 26–33 years reported giving supplements to their children. There was a significant difference in the proportion of women who reported that they are knowledgeable about rich food sources of iron between the mothers in the different age groups (*p* = 0.022), with the highest proportion of mothers in the age group of 26–33 years (92.9%).

Table 3 shows a significant difference in the types of food given to infants during the first 6 months according to the mother’s employment status (34.6% employed and 51.5% unemployed, respectively; *p* < 0.001). Moreover, a significantly higher proportion of unemployed mothers reported feeding their children compared to employed mothers (97.5% vs. 76.7%, respectively; *p* < 0.001). In addition, 65.4% of employed women gave supplements to their children in comparison to 49.4% of unemployed women (*p* = 0.002). There was no significant difference between employed and unemployed mothers in terms of the child’s age when breastfeeding was stopped. A higher number of unemployed mothers (43.9%) were found to continue breastfeeding after the children were older than 6 months compared to those who were employed (32.3%).

Table 3 also depicts feeding practices in terms of the maternal educational level. Although more mothers of lower education continued breastfeeding their children after 6 months (47.8% vs. 37.1%, respectively), a significantly higher percentage of mothers of lower education gave their children “other milk” (milk other than breastmilk or formula) (*p* = 0.035), most of whom introduced it between ≥6 and 11.9 months. Specifically, 56.5% of mothers with a university degree introduced other milk after their child’s first year of age compared to 45.7% of mothers with a high school education or less (*p* = 0.022). There was no significant difference in the proportion of mothers who reported that they are knowledgeable about food sources of iron according to their educational level.

Lastly, results from a multinomial logistic regression, performed so as to examine the odds of EBF compared to (i) the use of breastmilk and formula and to (ii) the use of formula, are shown in Table 4. As per the preliminary tabulations, the likelihood of using breastmilk and formula was significantly higher among women aged 25–33 years (OR: 1.7; 95% CI: 1.014–2.958) and 33+ years (OR: 2.2; 95% CI: 1.262–3.993) compared to younger women (18–25 years), as well as employed women compared to unemployed women (OR: 2.0; 95% CI: 1.298–3.220). No significant associations were found between women who were formula feeding compared to those practicing EBF. The model was adjusted for nationality, the type of delivery, and weeks of gestation.

### 3.4. Infants’ and Toddlers’ Intake of Fluids and Food Groups

Table 5 shows the frequency of weekly fluid intake among the total sample, including the infants and toddlers, during the past 4 weeks based on a qualitative FFQ. The daily intakes of animal milk and water were significantly higher among the toddlers (*p* < 0.001), with no significant difference in the intake of breastmilk and formula or soymilk between infants and toddlers.

**Figure 2:**
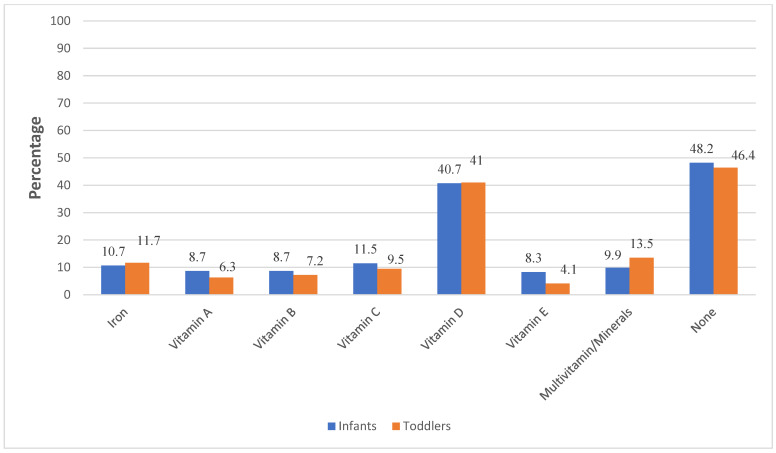
Prevalence of reported dietary supplement use among infants and toddlers.

Table 5 also shows the food intake (frequencies per day and per week) of the total number of children, infants, and toddlers in the study during the 4 weeks preceding the interview based on a qualitative FFQ. Overall, most of the infants and toddlers were not meeting the recommended daily or weekly frequencies for most foods. The weekly median intake of total fruits and vegetables per day among the toddlers was 3.3 (0.87, 6.0), while the intake of chips and sweets was 5.6 (2.5, 18.3). Our study population showed that the prevalence of vitamin and mineral supplements use among 6–12-month-old infants and 1–2.5-year-old toddlers was 51.8% and 53.6, respectively (overall 51.8%) (Figure 2). The most frequently reported supplement given to the infants and the toddlers was vitamin D, followed by vitamin/mineral supplements for toddlers.

### 3.5. Introduction of Food Sources of Iron and Use of Vitamin/Mineral Supplements

The timing of the introduction of the first animal-based protein foods into the children’s diets according to the knowledge of mothers about iron sources is presented in Figure S2 (Supplementary Materials). A significant percentage of mothers who reported being unaware of iron sources introduced their children to animal-based foods at or after 12 months of age (*p* = 0.024). Figure 2 shows the prevalence of vitamin and mineral supplement use among 6–12-month-old infants and 1–2.5-year-old toddlers in the study population. Overall, 52.6% of the children received vitamin/mineral dietary supplements (51.8% of infants and 53.6 of toddlers). The most frequently reported supplement given to the infants and the toddlers was vitamin D, followed by vitamin/mineral supplements for toddlers.

## 4. Discussion

We conducted a survey of mothers of children aged from 6 months to 2.5 years in four northern emirates of the UAE in order to assess child feeding practices. Although breastfeeding is rooted in the cultural and religious values of mothers in the UAE [6,38], our results showed that 46.7% of the infants were exclusively breastfed during the first 6 months. Significant differences in the types of feeding were found correlating with maternal age (*p* = 0.02) and employment status (*p* < 0.001) but not with educational level, with a higher proportion of younger and unemployed women exclusively breastfeeding. We also found that, at the time of the study, 22.1% of the infants and 8.1% of toddlers did not receive an animal source of iron, while 52.6% of the children were given vitamin/mineral supplements. Moreover, the frequency of the intake of nutrient-dense food groups was lower than the intake of sweets and savory snacks.

In the present study, less than 50% of the children in the four selected northern emirates of the UAE were exclusively breastfed. A recent prospective study conducted in the emirates of Abu Dhabi, Dubai, Sharjah, and Fujairah in the UAE found that only 26.7% of mothers were still exclusively breastfeeding their infants at the age of 6 months [39]. During the first 6 months, over half (53.3%) of the mothers in the present study reported using formula (alone or in combination with breastfeeding). In a previous study, although 93% of the women responded that they initially planned to breastfeed, the majority also planned to combine it with formula (7). Another recent survey conducted in the emirates of Abu Dhabi, Dubai, and Sharjah reported that, although 95% of infants were breastfed at some point in their lives, only 37% were EBF at 6 months of age [30]. Likewise, lower EBF rates have been recorded in different Middle Eastern countries: 18.9% of mothers in Qatar [40], 9.7% in Egypt [41], 1% in Jordan [12]), 28% in Saudi Arabia [42], and 32% in Lebanon [43]. Globally, 66.4% of infants in Iran were EBF [16], in Turkey 38.9% [44], and in Japan 37.4% [45], thus indicating that global EBF rates for the first 6 months of the child’s life are suboptimal.

Our study addressed gaps in research on the feeding practices of infants and toddlers in northern regions of the UAE, where relatively less information is available. We identified how maternal socio-demographics, such as age, education, and employment, are associated with the feeding of infants and toddlers. This study is the first of its kind to be conducted in the four northern regions of the UAE, as well as the first that included a large proportion of non-Emirati mothers. Most of the studies published from the UAE have focused on the other three emirates (Abu Dhabi, Dubai, and Sharjah) and included predominately mothers who were Emirati nationals.

An important factor to consider in regard to the promotion of breastfeeding is age. Based on our study results, although a higher percentage of younger women compared with women aged ≥26 years reported EBF (58.5%), most of the mothers aged 18–25 years stopped breastfeeding after 6 months. Based on a previous study, the most frequent reasons for which mothers stop breastfeeding are insufficient/perceived insufficient milk, infant feeding difficulties, and another pregnancy (7). Similarly, another study also found that more mothers aged <25 years began breastfeeding, but the mean months of the duration of breastfeeding were lower (7.2 months ± 5.1 compared to ≥9.7 months ± 7.0 among mothers ≥25 years (8)). Higher employment rates among this age group may be another possible reason.

Our results showed that a lower number of employed women provided their infants with EBF during the first 6 months. This finding correlates with previous studies conducted in the UAE [7,10,11]. A similar trend highlighting the impact of maternal employment on infant feeding has also been reported in other countries in the Middle East [13,15,40,46,47], the United States [18,20,48], Mexico [19], and the United Kingdom (UK) [49]. A recent systematic review and meta-analysis found that, across continents, the Middle East had the lowest total prevalence of breastfeeding, with a value of 10%, after mothers return to work [50]. Hence, employment seems to negatively impact infant breastfeeding practices, possibly due to the fact that women are not receiving enough support from healthcare providers or society, emotional stress, short periods of maternity leave, and challenges linked to merging breastfeeding with other maternal duties and tasks [10,13,15,39]. Methods for supporting employed mothers in maintaining breastfeeding, such as onsite childcare centers and educational activities, should be explored, considering that about 46% of females in the UAE were employed in 2020 [51].

A significantly higher proportion of mothers with a lower level of education in the present study introduced “other milk” before their child’s first year of age compared to those with a university education (*p* = 0.022). Research on the associations of maternal education and age with EBF, as well as the continuation of breastfeeding, has shown conflicting results [46]. Studies in Kuwait, Egypt, Iran, and Australia reported a positive association between maternal education level and the continuation of breastfeeding [13,14,16,17]. In our study, we found no significant difference between mothers in the three age groups in terms of when they stopped EBF, which differs from the findings of studies conducted in other Middle Eastern countries [12,14], as well as those of a study conducted in the UK [49]. According to a study in rural Egypt, mothers younger than 25 years of age maintained EBF for a longer period of time [14], while Radwan and colleagues [39] found that mothers aged 25–29.9 were significantly more likely to continue to breastfeed than those who were younger or older. A recent prospective study in the UAE identified perceived support from family and friends as a significant predictor of breastfeeding at 6 months [39]. Thus, a potential strategy for increasing EBF among mothers in the UAE is to increase the awareness of the importance of EBF in the mothers’ social networks, such as families.

Introducing solid foods at an appropriate time is a crucial factor in the healthy growth and development of infants [1]. In our study, 41.6% of mothers started introducing their infants to solid foods at less than 6 months of age. The early introduction of complementary foods is a matter of concern in the current study, since certain foods, such as dairy products and eggs, can initiate allergies [52]. Due to the hot weather conditions in the UAE, giving water to infants before 6 months of age is a common practice [7]. However, there is no requirement for supplementary fluids for breastfed infants, even in hot climates, since breastmilk alone can fulfill their water requirements [53].

A range of animal proteins must be initiated, starting at 6 months of age, to prevent iron deficiency [1]. In our study, 88.8% of the mothers reported knowledge about food sources of iron, with those reporting a low knowledge of this mineral showing a delay in the introduction of the food sources of at least 12 months. In a study of 276 infants and toddlers aged 0–23.9 months in the UAE, the usual intake of iron was 47% among infants and 11% among toddlers, respectively, which is below the estimated average requirement [30]. Our findings highlight the need to increase mothers’ awareness about introducing their infants to rich sources of iron. The low daily intakes of dairy, fruit, and vegetables among both the infants and the toddlers in the present study are consistent with the findings of a recent study conducted in the other emirates of the UAE [54].

There is limited information on dietary supplement use and the motivators of giving nutrition supplements to young children in the UAE. In the present study, a significant number of mothers (65.4%) gave supplements to their children, especially those who were employed. Other UAE-based studies showed that the rate of supplementation varied between 14% and 53% [7,8]. In a more recent study conducted in Abu Dhabi, Dubai, and Sharjah, vitamin/mineral supplement use was calculated at 59% [30]. One of the main reasons for giving supplements to infants and toddlers was the perception of the inadequacy of breastmilk [8]. In a nationally representative sample of infants and children in the US, dietary supplements use was calculated at 18.2% [55]. There is a need for additional studies exploring the motivators of supplement use, as well as nutrient contributions from dietary supplements, among infants and toddlers in the UAE. The results of our study are important for the UAE and may have implications for other countries in the Arab Gulf region with a similar socio-economic status. To the best of our knowledge, this is the first study on child feeding practices among mothers with children aged between 6 months and 2.5 years conducted in the four northern emirates in the UAE, a region where information on child feeding practices is scarce. Moreover, in this study, we considered maternal socio-demographic factors, such as age, education, and employment status, and controlled potential confounding factors, such as the mode of delivery and weeks of gestation. Furthermore, the mothers included in the study were both UAE nationals and non-nationals; thus, a more representative proportion of the general reproductive population of mothers in the UAE was studied, which has not been reported in other studies conducted in the UAE. However, this study has some limitations, with one being a recall bias due to the nature of the data collection in this retrospective study using qualitative FFQs, particularly regarding the assessment of the introduction of complementary foods and liquids. This recall bias may have resulted in the under- or overestimation of the food intake and actual breastfeeding practices. Moreover, our study did not investigate the reasons leading to the early stopping of EBF, the early introduction of complementary foods, and the use of supplements. Future studies addressing the above-mentioned limitations should be conducted at the national level.

## 5. Conclusions

This study addressed gaps in research on the feeding practices of infants and toddlers in northern regions of the UAE, where relatively less information is available. We identified how maternal socio-demographics, such as age, education, and employment status, are associated with the feeding of infants and toddlers. Less than 50% of the mothers exclusively breastfed their infants during the first 6 months of life. Moreover, the frequency of the intake of nutrient-dense food groups among the children was sub-optimal. Therefore, it is necessary to provide mothers in the UAE with the information needed in regard to healthy infant and toddler feeding practices, as well as the support required to maintain breastfeeding.

## Figures and Tables

**Table 1:** Maternal socio-demographic characteristics.

Maternal Characteristic	Total Population (n = 475)
Age group, n (%)	
18–25 years	106 (22.3)
26–33 years	227 (47.8)
34+ years	142 (29.9)
Educational level, n (%)	
Basic/high school	157 (33.1)
University level	318 (66.9)
Professional status, n (%)	
Employed (Part-time, full-time, or entrepreneur)	342 (72.0
Unemployed	133 (28.0)
Nationality, n (%)	
UAE national	262 (55.2)
UAE non-national	213 (44.8)
Sources of information used for child feeding	
Health professionals	148 (31.2)
Family/friends	137 (28.8)
Social media	131 (27.6)
Other	59 (12.4)

**Table 2:** Child feeding practices by maternal age category.

Variable	Total n = 475	18–25 Years n = 106, 22.3%	26–33 Years n = 227, 47.8%	34+ Years n = 142, 29.9%	*p*-Value
Type of feeding for first 6 months					0.020
Exclusive breastfeeding (EBF)	222 (46.7)	62 (58.5)	102 (44.9)	58 (40.9)	
Breastmilk and formula	208 (43.8)	32 (30.2)	103 (45.4)	73 (51.4)	
Only formula	45 (9.5)	12 (11.3)	22 (9.7)	11 (7.8)	
Age when breastfeeding stopped (n = 339) *					0.272
<6 months	181 (53.4)	42 (61.8)	88 (52.4)	51 (49.5)	
≥6 months	158 (46.6)	26 (38.2)	80 (47.6)	52 (50.5)	
Age when child was given milk other than breastmilk or formula					0.252
<6 months	89 (22.9)	20 (22.0)	37 (20.2)	32 (27.8)	
≥6 months–11.9 months	94 (24.2)	28 (30.8)	41 (22.4)	25 (21.7)	
≥1 year	206 (52.9)	43 (47.3)	105 (57.4)	58 (50.4)	
Age when started solid food					0.964
<6 months	182 (41.6)	38 (43.2)	86 (40.6)	58 (42.0)	
≥6 months–<1 year	234 (53.4)	47 (53.4)	114 (53.8)	73 (52.9)	
≥1 year	22 (5.0)	3 (3.4)	12 (5.7)	7 (5.1)	
Age for introducing animal-based protein					0.156
6–7 months	89 (18.7)	20 (18.9)	38 (16.7)	31 (21.8)	
8–9 months	137 (28.8)	32 (30.2)	75 (33.0)	30 (21.1)	
10–11 months	79 (16.6)	23 (21.7)	34 (15.0)	22 (15.5)	
≥12 months	89 (18.7)	13 (12.3)	45 (19.8)	31 (21.8)	
Don’t give any	81 (17.1)	18 (17.0)	35 (15.4)	28 (19.7)	
Type of first animal protein given					0.017
Beef	9 (1.9)	3 (2.8)	3 (1.3)	3 (2.1)	
Chicken	143 (30.1)	27 (25.5)	70 (30.8)	46 (32.4)	
Egg yolk	131 (27.6)	35 (33.0)	66 (29.1)	30 (21.1)	
Fish	31 (6.5)	13 (12.3)	13 (5.7)	5 (3.5)	
Liver	4 (0.8)	0 (0)	2 (0.9)	2 (1.4)	
Whole egg	83 (17.5)	8 (7.6)	44 (19.4)	31 (21.8)	
None yet	74 (15.6)	20(18.9)	29(12.8)	25(17.6)	
Infants	56(22.1)	14(20.0)	23(20.2)	19(27.5)	
Toddlers	18(8.1)	6 (16.7)	6 (5.3)	6 (8.2)	
Use of salt or honey in child’s food, % yes	210 (44.2)	44 (41.5)	95 (41.9)	71 (50.0)	0.252
Infants	89 (35.2)	23 (32.9)	37 (32.5)	29 (42.0)	0.376
Toddlers	121 (51.5)	21 (58.3)	58 (51.3)	42 (17.8)	0.624
Child’s food prepared usually, %					0.406
Home	467 (98.3)	105 (99.1)	225 (99.1)	137 (96.5)	
Family/friends	4 (0.8)	1 (0.9)	0 (0)	3 (2.1)	
Restaurant/take away	2 (0.4)	0 (0)	1 (0.4)	1 (0.7)	
Other	2 (0.4)	0 (0)	1 (0.4)	1 (0.7)	
Who feeds the child					
Mother	435 (91.6)	99 (93.4)	213 (93.8)	123 (86.6)	0.040
Father	5 (1.1)	3 (2.8)	0 (0)	2 (1.4)	
Grandparent	6 (1.3)	1 (0.9)	2 (0.9)	3 (2.1)	
Nanny	27 (5.7)	3 (2.8)	10 (4.4)	14 (9.9)	
Other	2 (0.4)	0 (0)	2 (0.9)	0 (0)	
Use of supplements	250 (52.6)	43 (40.6)	132 (58.2)	75 (52.8)	0.011
Infants	131 (51.8)	28 (40.0)	71 (62.3)	32 (51.8)	0.008
Toddlers	119 (53.6)	15 (41.7)	61 (54.0)	43 (58.9)	0.235
Knowledge of iron-rich foods	422 (88.8)	89 (84.0)	211 (92.9)	122 (85.9)	0.022

Assessed using the chi-squared test at α = 5%; EBF: exclusive breastfeeding. * A total of 136 of 475 were still breastfeeding.

**Table 3:** Child feeding practices by maternal employment status and educational level (n, %).

Variable	Unemployed 342 (72) n (%)	Employed 133 (28) n (%)	*p*-Value	≤High School 106 (22.3) n (%)	University 369 (77.7) n (%)	*p*-Value
Type of food over first 6 months			<0.001			0.363
Exclusive breastfeeding (EBF)	176 (51.5)	46 (34.6)		80 (51.0)	142 (44.7)	
Breastmilk and Formula	131 (38.3)	77 (57.9)		65 (41.4)	143 (45.0)	
Only formula	35 (10.2)	10 (7.5)		12 (7.6)	33 (10.4)	
Child’s age when BF stopped			0.272			0.598
<6 months	119 (50.2)	62 (60.8)		61 (55.5)	120 (52.4)	
≥6 months	118 (49.8)	40 (39.2)		49 (44.5)	109 (47.6)	
Continued breastfeeding after 6 months	150 (43.9)	43 (32.3)	0.022	75 (47.8)	118 (37.1)	0.026
Gave child milk other than breastmilk or formula	129 (37.7%)	51 (38.4%)	0.899	70 (44.6)	110 (34.6)	0.035
Age when “other milk” was given (does not include breastmilk or formula)			0.066			0.022
<6 months	57 (20.7)	32 (28.1)		28 (21.7)	61 (23.5)	
≥6 months–11.9 months	62 (22.6)	32 (28.1)		42 (32.6)	52 (20.0)	
≥1 year	156 (56.7)	50 (43.9)		59 (45.7)	147 (56.5)	
Age when started solid food			0.271			0.791
<6 months	130 (41.5)	52 (41.6)		59 (39.3)	123 (42.7)	
≥6 months–<1 year	164 (52.4)	70 (56.0)		83 (55.3)	151 (52.4)	
≥1 year	19 (6.1)	3 (2.4)		8 (5.3)	14 (4.9)	
Age when animal-based protein was given			0.064			0.312
6–7 months	57 (16.7)	32 (24.1)		34 (21.7)	55 (17.3)	
8–9 months	92 (26.9)	45 (33.8)		37 (23.6)	100 (31.5)	
10–11 months	62 (18.1)	17 (12.8)		30 (19.1)	49 (15.4)	
≥12 months	71 (20.8)	18 (13.5)		27 (17.2)	62 (19.5)	
Do not give any	60 (17.5)	21 (15.8)		29 (18.5)	52 (16.4%)	
Infants	47 (24.6)	15 (24.2)	0.012	23 (23.5)	39 (25.2)	0.533
Toddlers	13 (8.6)	6 (8.5)	0.793	6 (10.2)	13 (8.0)	0.588
First animal protein given			0.183			0.166
Beef	9 (2.6)	0 (0)		2 (1.3)	7 (2.2)	
Chicken	102 (29.8)	41 (30.8)		49 (31.2)	94 (29.6)	
Egg yolk	86 (25.2)	45 (33.8)		36 (22.9)	95 (29.9)	
Fish	24 (7.0)	7 (5.3)		15 (9.5)	16 (5.0)	
Liver	2 (0.6)	2 (1.5)		3 (1.9)	1 (0.3)	
Whole egg	64 (18.7)	19 (14.3)		26 (16.6)	57 (17.9)	
None yet	55 (16.1)	19 (14.3)		26 (16.6)	48 (15.1)	
Use of salt or honey in child’s food, n = 210 (%)	149 (43.6)	61 (45.9)	0.651	77 (49.0)	133 (41.8)	0.136
Infants	64 (33.5)	25 (40.3)	0.329	40 (40.8)	49 (31.6)	0.135
Toddlers	85 (56.3)	36 (50.7)	0.436	37 (62.7)	84 (51.5)	0.140
Who feeds the child?			<0.001			0.066
Mother	333 (97.4)	102 (76.7)		151 (96.2)	284 (89.3)	
Father	3 (0.9)	2 (1.5)		0 (0)	5 (1.6)	
Grandparent	1 (0.3)	5 (3.8)		0 (0)	6 (1.9)	
Nanny	5 (1.5)	22 (16.5)		5 (3.2)	22 (6.9)	
Other	0 (0)	2 (1.5)		1 (0.6)	1 (0.3)	
Use of supplements, Yes (%)	163 (47.7)	87 (65.4)	0.001	61 (57.6)	189 (51.2)	0.250
Infants	88 (46.1)	43 (69.4)	0.001	58 (59.2)	73 (47.1)	0.061
Toddlers	75 (49.7)	44 (62.0)	0.086	3 (37.5)	116 (54.2)	0.477
Knowledge of iron-rich foods, Yes (%)	304 (88.9)	118 (88.7)	0.959	138 (87.9)	284 (89.3)	0.646

Assessed using the chi-squared test at α = 5% or Fisher’s exact test for cell frequency values of n ≤ 5. EBF: exclusive breastfeeding.

**Table 4:** Multinomial logistic regression of the feeding type received during the first 6 months compared to mixed feed and formula alone.

Variables	Odds Ratio	95% Confidence Interval		*p* Value *
Exclusive breastfeeding	(base outcome)			
			
Breastmilk and formula				
Age category ^1^				
25–33 years old	1.7	1.014–2.958		0.044
33+ years old	2.2	1.262–3.993		0.006

Educational level ^2^	1.1	0.698–1.672		0.729
Employed vs. unemployed	2.0	1.298–3.220		0.002
Nationality ^3^				
Arab	0.9	0.607–1.445		0.768
Non-Arab	0.7	0.222–2.003		0.470
Method of delivery	1.2	0.728–1.863		0.526
Weeks of gestation	0.9	0.583–1.467		0.739

**Only formula**				
Age category ^1^				
25–33	0.8	0.367–1.913		0.674
33+	0.8	0.307–1.994		0.607
Educational level ^2^	1.3	0.627–2.875		0.449
Employed vs. unemployed	1.0	0.437–2.170		0.949
Nationality ^3^				
Arab	1.7	0.819–3.517		0.155
Non-Arab	0.7	0.079–6.375		0.761
Method of delivery	1.9	0.901–3.821		0.094
Weeks of gestation	1.2	0.548–2.584		0.660

* Significance level set at alpha = 5%. Model adjusted for the method of delivery (caesarean section, normal birth), nationality, and weeks of gestation. 1: Women aged 18–25 years, the baseline for the age category dummy variable. 2: University level compared to ≤high school level of education. 3: Emirati nationality, the bassline comparator.

**Table 5:** Frequency of fluid and food group intake (in servings per day or week) in the total population studied, including infants and toddlers.

Variable	Total Children	Infants (n = 253)	Toddlers (n = 222)	*p*-Value
Daily intake, median (25–75% range)				
Breastmilk	0.64 (0, 2.0)	0.64 (0.1, 2.0)	0.64 (0, 2.0)	0.732
Formula/soya milk	0.29 (0, 2.0)	0.29 (0, 2.0)	0.64 (0, 2.0)	0.404
Animal milk	0.1 (0, 0.64)	0 (0, 0.29)	0.29 (0. 2.0)	<0.001
Water	2.0 (0.3, 4.0)	0.64 (0.1, 2.0)	2.0 (0.64, 4.0)	<0.001
Weekly intake, median (25–75% range)				
100% fruit or vegetable juice	2.0 (0, 14)	0.5 (0, 4.5)	2.0 (0.5, 14)	<0.001
Ready to drink (packaged) fruit or vegetable juice	2.0 (0.5, 14)	2.0 (0.5, 14)	2.0 (0.5, 14)	0.249
Soft drinks	0 (0, 4.5)	0 (0, 4.5)	0 (0, 14)	0.477
Food groups, daily ^1^ intake, median (25–75% range)				
Grains and cereal	2.9 (1.3, 6.1)	2.6 (1.2, 5.3)	3.4 (1.3, 8.0)	0.002
Dairy	0.3 (0.07, 2.0)	0.07 (0, 0.64)	0.64 (0.29, 2.0)	<0.001
Tubers	0.3 (0.07, 2.0)	0.3 (0.07, 2.0)	0.3 (0.29, 2.0)	0.027
Vitamin-A-rich fruit & vegetables	0.64 (0.29, 2.0)	0.64 (0.1, 2.0)	0.64 (0.29, 2.0)	0.003
Vegetables (other)	0.64 (0.07, 2.0)	0.29 (0.07, 0.64)	0.64 (0.29, 2.0)	
Fruit (other)	0.29 (0.07, 0.64)	0.29 (0.07, 0.64)	0.29 (0.64, 2.0)	<0.001
Total fruits and vegetables	1.92 (0.87, 5.28)	1.57 (0.43, 3.28)	3.28 (0.87, 6.0)	<0.001
Weekly ^2^ intake, median (25–75% range)				
Eggs	2.0 (0.5, 4.5)	2.0 (0, 4.5)	2.0 (2.0, 14.0)	<0.001
Pulses/legumes	2.0 (0.5, 14.0)	2.0 (0.5, 4.5)	2.0 (2.0, 14.0)	0.054
Red meat, organ meats	2.0 (0, 4.5)	0.5 (0, 4.5)	0.5 (2.0, 4.5)	<0.001
Poultry	2.0 (0.5, 4.5)	2.0 (0, 4.5)	2.0 (2.0, 14.0)	<0.001
Fish	2.0 (0.5, 4.5)	0.5 (0, 4.5)	2.0 (0.5, 4.5)	<0.001
Sweets (all baked goods and desserts)	2.0 (0.6, 4.4)	1.5 (0.5, 3.2)	2.6 (0.9, 4.9)	<0.001
Chips and savory snacks	2.0 (0, 4.5)	0.5 (0, 4.5)	2.0 (0.5, 14)	<0.001
Take away food	0.5 (0, 2.0)	0.5 (0, 2.0)	2.0 (0, 4.5)	<0.001

^1^ Daily or ^2^ weekly median intake (25–75% range) based on the food frequency questionnaire. Significance at a = 5%, assessed with Kruskal–Wallis test. Grains and cereals, including rice, cold and hot cereal, bread/crackers, rice, and pasta. Dairy: all non-milk products (including cheese and yogurt). Sweets: biscuits, sweet snacks, sweet jelly, spreads, and honey.

## Data Availability

The dataset used to prepare this analysis is available from the corresponding author.

## Supplementary Materials

The following supporting information can be downloaded at: https://www.mdpi.com/article/10.3390/nu14183719/s1, Table S1: Food Frequency Questionnaire; Figure S2: Percentage of mothers that have given animal-based protein to their children and timing by knowledge of food sources of iron.

## References

[1] World Health Organization-Infant and Young Child Feeding. https://www.who.int/news-room/fact-sheets/detail/infant-and-young-child-feeding.

[2] UNICEF-IRC-The First 1000 Days of Life: The Brain’s Window of Opportunity. https://www.unicef-irc.org/article/958-the-first-1000-days-of-life-the-brains-window-of-opportunity.html.

[3] Kelishadi R., Farajian S. (2014). The Protective Effects of Breastfeeding on Chronic Non-Communicable Diseases in Adulthood: A Review of Evidence. Adv. Biomed. Res..

[4] Souza L.L., de Moura E.G., Lisboa P.C. (2020). Does Early Weaning Shape Future Endocrine and Metabolic Disorders? Lessons from Animal Models. J. Dev. Orig. Health Dis..

[5] Bagci Bosi A.T., Eriksen K.G., Sobko T., Wijnhoven T.M.A., Breda J. (2016). Breastfeeding Practices and Policies in WHO European Region Member States. Public Health Nutr..

[6] Taha Z. (2017). Trends of Breastfeeding in the United Arab Emirates (UAE). Arab. J. Nutr. Exerc. (AJNE).

[7] Gardner H., Green K., Gardner A. (2015). Infant Feeding Practices of Emirati Women in the Rapidly Developing City of Abu Dhabi, United Arab Emirates. Int. J. Environ. Res. Public Health.

[8] Radwan H. (2013). Patterns and Determinants of Breastfeeding and Complementary Feeding Practices of Emirati Mothers in the United Arab Emirates. BMC Public Health.

[9] Taha Z., Garemo M., Nanda J. (2020). Complementary Feeding Practices among Infants and Young Children in Abu Dhabi, United Arab Emirates. BMC Public Health.

[10] Taha Z., Hassan A., Wikkeling Scott L., Papandreou D. (2021). Factors Associated with Delayed Initiation and Cessation of Breastfeeding Among Working Mothers in Abu Dhabi, the United Arab Emirates. Int. J. Women’s Health.

[11] Hameed N., Tatari H.A. (2015). Maternal Factors Hindering Successful Breastfeeding in Al Ain City, United Arab Emirates. J. Women’s Health Care.

[12] Abuidhail J., Al-Modallal H., Yousif R., Almresi N. (2014). Exclusive Breast Feeding (EBF) in Jordan: Prevalence, Duration, Practices, and Barriers. Midwifery.

[13] Dashti M., Scott J.A., Edwards C.A., Al-Sughayer M. (2014). Predictors of Breastfeeding Duration among Women in Kuwait: Results of a Prospective Cohort Study. Nutrients.

[14] Shafei A.M.H.E., Labib J.R. (2014). Determinants of Exclusive Breastfeeding and Introduction of Complementary Foods in Rural Egyptian Communities. Glob. J. Health Sci..

[15] Al-Ruzaihan S.A., Al-Ghanim A.A., Bu-Haimed B.M., Al-Rajeh H.K., Al-Subaiee W.R., Al-Rowished F.H., Badger-Emeka L.I. (2017). Effect of Maternal Occupation on Breast Feeding among Females in Al-Hassa, Southeastern Region of KSA. J. Taibah Univ. Med. Sci..

[16] Veghari G., Mansourian A., Abdollahi A. (2011). Breastfeeding Status and Some Related Factors in Northern Iran. Oman Med. J..

[17] Daly A., Pollard C.M., Phillips M., Binns C.W. (2014). Benefits, barriers and enablers of breastfeeding: Factor analysis of population perceptions in Western Australia. PLoS ONE.

[18] Mirkovic K.R., Perrine C.G., Scanlon K.S., Grummer-Strawn L.M. (2014). Maternity Leave Duration and Full-Time/Part-Time Work Status Are Associated with US Mothers’ Ability to Meet Breastfeeding Intentions. J. Hum. Lact..

[19] Rivera-Pasquel M., Escobar-Zaragoza L., González de Cosío T. (2015). Breastfeeding and maternal employment: Results from three national nutritional surveys in Mexico. Matern. Child Health J..

[20] Dagher R.K., McGovern P.M., Schold J.D., Randall X.J. (2016). Determinants of Breastfeeding Initiation and Cessation among Employed Mothers: A Prospective Cohort Study. BMC Pregnancy Childbirth.

[21] Scott J., Ahwong E., Devenish G., Ha D., Do L. (2019). Determinants of Continued Breastfeeding at 12 and 24 Months: Results of an Australian Cohort Study. Int. J. Environ. Res. Public Health.

[22] Carter R.C., Jacobson J.L., Burden M.J., Armony-Sivan R., Dodge N.C., Angelilli M.L., Lozoff B., Jacobson S.W. (2010). Iron Deficiency Anemia and Cognitive Function in Infancy. Pediatrics.

[23] Yang W., Liu B., Gao R., Snetselaar L.G., Strathearn L., Bao W. (2021). Association of Anemia with Neurodevelopmental Disorders in a Nationally Representative Sample of US Children. J. Pediatr..

[24] Obbagy J.E., English L.K., Psota T.L., Wong Y.P., Butte N.F., Dewey K.G., Fox M.K., Greer F.R., Krebs N.F., Scanlon K.S. (2019). Complementary Feeding and Micronutrient Status: A Systematic Review. Am. J. Clin. Nutr..

[25] WHO Anemia. https://www.who.int/health-topics/anaemia#tab=tab_1.

[26] Kumar D., Qureshi Z.N., Albadwawi M.S. (2019). Iron Deficiency Anemia in Infants of Hatta Suburb-UAE. Int. Arch. Nurs. Health Care.

[27] Faysal W., Zaidi A.R.Z., Al-Abdi S., Alhumaid S., AlShehery M.Z., Al Mutair A. (2020). Hospital-Based Prevalence of Iron Deficiency Anemia among Pre-School Children in Dubai. Cureus.

[28] Nasreddine L.M., Kassis A.N., Ayoub J.J., Naja F.A., Hwalla N.C. (2018). Nutritional status and dietary intakes of children amid the nutrition transition: The case of the Eastern Mediterranean Region. Nutr Res..

[29] Abdulrazzaq Y.M., Nagelkerke N., Abdulla S., Belhaj G. (2016). Nutrient intake of infants and toddlers in the United Arab Emirates: The Feeding Infants and Toddlers Study. EMHJ-East. Mediterr. Health J..

[30] Cheikh Ismail L., Al Dhaheri A.S., Ibrahim S., Ali H.I., Chokor F.A.Z., O’Neill L.M., Mohamad M.N., Kassis A., Ayesh W., Kharroubi S. (2022). Nutritional Status and Adequacy of Feeding Practices in Infants and Toddlers 0-23.9 Months Living in the United Arab Emirates (UAE): Findings from the Feeding Infants and Toddlers Study (FITS) 2020. BMC Public Health.

[31] Federal Competitiveness and Statistics Authority-Statistics by Subject. https://fcsa.gov.ae/en-us/Pages/Statistics/Statistics-by-Subject.aspx#/%3Fyear=&folder=Demography%20and%20Social/Vital%20Statistics/Births%20and%20Deaths&subject=Demography%20and%20Social.

[32] Villar J., Altman D.G., Purwar M., Noble J.A., Knight H.E., Ruyan P., Cheikh Ismail L., Barros F.C., Lambert A., Papageorghiou A.T. (2013). The Objectives, Design and Implementation of the INTERGROWTH-21st Project. BJOG.

[33] The International Fetal and Newborn Growth Consortium for the 21st Century (INTERGROWTH-21st)-Study Protocol and Other Project Documents. https://www.intergrowth21.org.uk/protocol.aspx?lang=1.%20.

[34] Cheikh Ismail L., Giuliani F., Bhat B.A., Bishop D., Papageorghiou A.T., Ochieng R., Puglia F., Altman D.G., Maia-Schlüssel M., Noble J.A. (2016). Preterm Feeding Recommendations Are Achievable in Large-Scale Research Studies. BMC Nutr..

[35] Subar A.F., Dodd K.W., Guenther P.M., Kipnis V., Midthune D., McDowell M., Tooze J.A., Freedman L.S., Krebs-Smith S.M. (2006). The Food Propensity Questionnaire: Concept, Development, and Validation for Use as a Covariate in a Model to Estimate Usual Food Intake. J. Am. Diet Assoc..

[36] Kittisakmontri K., Fewtrell M., Roekworachai K., Phanpong C., Lanigan J. (2019). Complementary Feeding: Attitudes, Knowledge and Practices of Urban Families in Northern Thailand. Nutr. Diet.

[37] StataCorp (2015). Stata Statistical Software: Release 14.

[38] Shaikh U., Ahmed O. (2006). Islam and infant feeding. Breastfeed Med..

[39] Radwan H., Fakhry R., Metheny N., Baniissa W., Faris M.A.I.E., Obaid R.S., Al Marzooqi S., Al Ghazal H., ElHalik M., Dennis C.-L. (2021). Prevalence and Multivariable Predictors of Breastfeeding Outcomes in the United Arab Emirates: A Prospective Cohort Study. Int. Breastfeed. J..

[40] Al-Kohji S., Said H.A., Selim N.A. (2012). Breastfeeding Practice and Determinants among Arab Mothers in Qatar. Saudi Med. J..

[41] Al Ghwass M.M.E., Ahmed D. (2011). Prevalence and Predictors of 6-Month Exclusive Breastfeeding in a Rural Area in Egypt. Breastfeed Med..

[42] Alyousefi N.A. (2021). Determinants of Successful Exclusive Breastfeeding for Saudi Mothers: Social Acceptance Is a Unique Predictor. Int. J. Environ. Res. Public Health.

[43] Issa C., Hobeika M., Salameh P., Zeidan R.K., Mattar L. (2019). Longer Durations of Both Exclusive and Mixed Breastfeeding Are Associated with Better Health in Infants and Toddlers. Breastfeeding Rev..

[44] Yılmaz E., Doğa Öcal F., Vural Yılmaz Z., Ceyhan M., Kara O.F., Küçüközkan T. (2017). Early Initiation and Exclusive Breastfeeding: Factors Influencing the Attitudes of Mothers Who Gave Birth in a Baby-Friendly Hospital. Turk. J. Obstet. Gynecol..

[45] Inano H., Kameya M., Sasano K., Matsumura K., Tsuchida A., Hamazaki K., Inadera H., Hasegawa T. (2021). Factors Influencing Exclusive Breastfeeding Rates until 6 Months Postpartum: The Japan Environment and Children’s Study. Sci. Rep..

[46] Alzaheb R.A. (2017). Factors Influencing Exclusive Breastfeeding in Tabuk, Saudi Arabia. Clin. Med. Insights Pediatr..

[47] Khasawneh W., Khasawneh A.A. (2017). Predictors and Barriers to Breastfeeding in North of Jordan: Could We Do Better?. Int. Breastfeed. J..

[48] Attanasio L., Kozhimannil K.B., McGovern P., Gjerdingen D., Johnson P.J. (2013). The impact of prenatal employment on breastfeeding intentions and breastfeeding status at 1 week postpartum. J. Hum. Lact..

[49] Oakley L.L., Henderson J., Redshaw M., Quigley M.A. (2014). The Role of Support and Other Factors in Early Breastfeeding Cessation: An Analysis of Data from a Maternity Survey in England. BMC Pregnancy Childbirth.

[50] Dutheil F., Méchin G., Vorilhon P., Benson A.C., Bottet A., Clinchamps M., Barasinski C., Navel V. (2021). Breastfeeding after Returning to Work: A Systematic Review and Meta-Analysis. Int. J. Environ. Res. Public Health.

[51] The World Bank-Labor Force Participation Rate, Female (% of Female Population Ages 15+) (Modeled ILO Estimate)-United Arab Emirates | Data. https://data.worldbank.org/indicator/SL.TLF.CACT.FE.ZS?locations=AE.

[52] Nuzzi G., Di Cicco M.E., Peroni D.G. (2021). Breastfeeding and Allergic Diseases: What’s New?. Children.

[53] Sachdev H.P., Krishna J., Puri R.K., Satyanarayana L., Kumar S. (1991). Water Supplementation in Exclusively Breastfed Infants during Summer in the Tropics. Lancet.

[54] Nassreddine L.M., Naja F.A., Hwalla N.C., Ali H.I., Mohamad M.N., Chokor F.A.Z.S., Chehade L.N., O’Neill L.M., Kharroubi S.A., Ayesh W.H. (2022). Total Usual Nutrient Intakes and Nutritional Status of United Arab Emirates Children (<4 Years): Findings from the Feeding Infants and Toddlers Study (FITS) 2021. Curr. Dev. Nutr..

[55] Gahche J.J., Herrick K.A., Potischman N., Bailey R.L., Ahluwalia N., Dwyer J.T. (2019). Dietary Supplement Use among Infants and Toddlers Aged <24 Months in the United States, NHANES 2007–2014. J. Nutr..

