# Supplementary material for: Feeding Practices of Infants and Toddlers by Their Mothers in Selected Northern Emirates of the United Arab Emirates
Source: Nutrients. 2022 Sep 9;14(18):3719. doi: 10.3390/nu14183719 (PMC9501191; doi:10.3390/nu14183719)
Supplement: Supplementary file 1 [file nutrients-14-03719-s001.zip › Table -S1-FFQ 0.5-2.5 year old infants and toddlers.pdf]

Please select how often your child had each item in the past 28 days. (choose one box) {Food Frequency Questionnaire (FFQ)}

Mark only one oval per row.

	Never	1-3 times/month	1-3 times/week	>3 times/week	1-3 times/day	>3 times/day
Breast milk						
Formula/soya milk						
Animal milk (for example: camel's milk, cows, goats, other)						
Fruit/vegetable juice, packaged						
Fruit/vegetable juice, freshly squeezed						
Soft drinks						
Water						
Soup						
Dairy products (e.g. yogurt, cheese)						
Cooked cereals (e.g. porridge)						
Cold cereals (e.g. cornflakes)						
Vitamin A -rich fruit/veg (e.g.						

carrot, spinach)						
Other fruits						
Other vegetables, (does not include potatoes and other tubers)						
Grains (e.g. rice)						
Legumes (e.g. beans, pulses)						
Pasta/ noodles						
Tubers (e.g. potatoes)						
Bread/crackers						
Biscuits/sweet snacks						
Chips/savoury snacks						
sweet jelly						
Honey						
Egg						
Red/organ meats (e.g. beef, lamb, pork, liver)						
Fish						
Poultry (e.g. chicken)						
Take-away						

food (e.g. pizza)						
Spreads (e.g. nutella, peanut butter)/oils	◯	◯	◯	◯	◯	◯

Thank for your participation

This content is neither created nor endorsed by Google.

Google Forms
