# Supplementary material for: Feeding Practices of Infants and Toddlers by Their Mothers in Selected Northern Emirates of the United Arab Emirates
Source: Nutrients. 2022 Sep 9;14(18):3719. doi: 10.3390/nu14183719 (PMC9501191; doi:10.3390/nu14183719)
Supplement: Supplementary file 1 [file nutrients-14-03719-s001.zip › Figure - S2-Introdction of animal protein by mother's knowledge of iron sources.pdf]

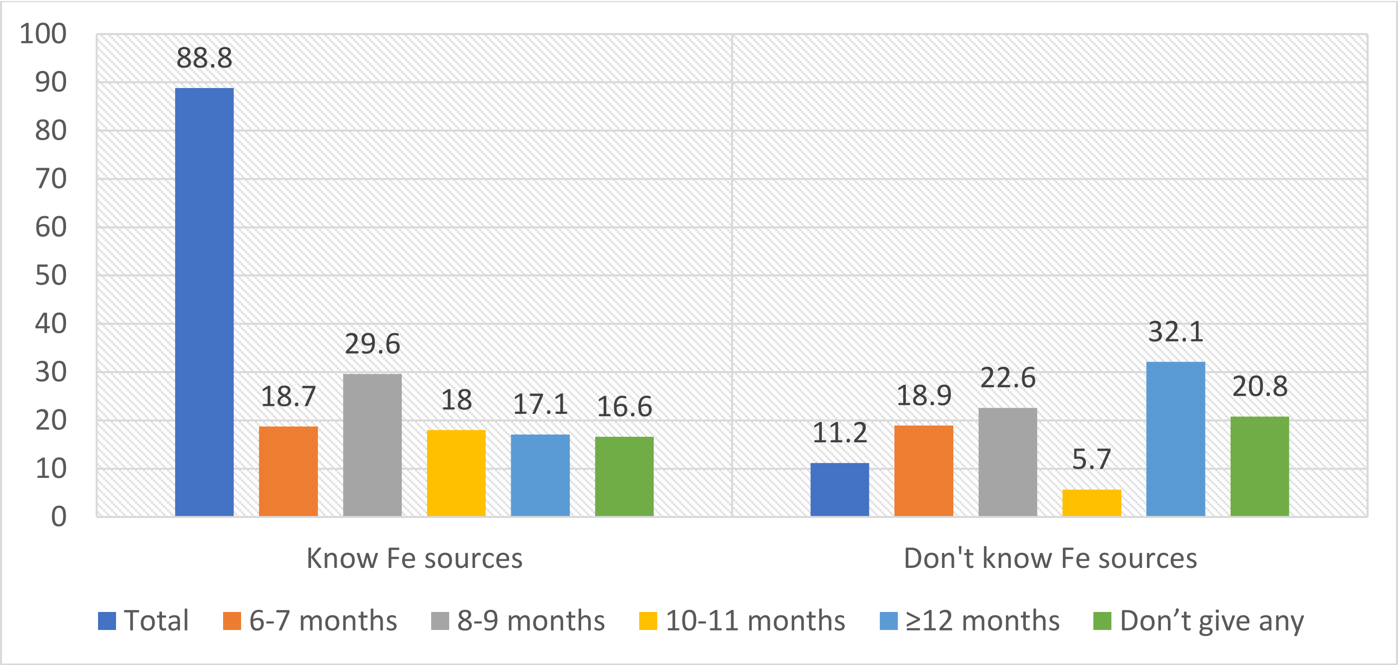

Figure, S2: Percentage of mothers that have given animal-based protein to their children and timing by knowledge of food sources of iron. p=0.024, assessed using chi-square test at α=5%; Fe= Iron
